# Association of Long-term Oncologic Prognosis With Minimal Access Breast Surgery vs Conventional Breast Surgery

**DOI:** 10.1001/jamasurg.2022.4711

**Published:** 2022-10-05

**Authors:** Andi Wan, Yan Liang, Li Chen, Shushu Wang, Qiyun Shi, Wenting Yan, Xiaozhen Cao, Ling Zhong, Linjun Fan, Peng Tang, Guozhi Zhang, Siyi Xiong, Cheng Wang, Zhen Zeng, Xiujuan Wu, Jun Jiang, Xiaowei Qi, Yi Zhang

**Affiliations:** 1 Department of Breast and Thyroid Surgery, Southwest Hospital, the First Affiliated Hospital of the Army Military Medical University, Chongqing, China

## Abstract

**Importance:**

Minimal access breast surgery (MABS) has been used in breast cancer management. However, long-term prognostic data associated with MABS vs conventional breast surgery (CBS) are lacking.

**Objective:**

To investigate long-term therapeutic outcomes associated with MABS vs CBS for breast cancer management.

**Design, Setting, and Participants:**

In this single-center retrospective cohort study, 9184 individuals were assessed for inclusion. After exclusions, 2412 adult female individuals were included who were diagnosed with stage 0 to III breast cancer, underwent unilateral breast surgery between January 2004 and December 2017, and had no distant metastasis or history of severe underlying disease. Propensity score matching was performed to minimize selection bias. Data were analyzed from January 1, 2004, to December 31, 2019.

**Exposures:**

MABS or CBS.

**Main Outcomes and Measures:**

Data on demographic and tumor characteristics and long-term outcomes were collected and analyzed.

**Results:**

This study included 2412 patients (100% female; median [IQR] age, 44 [40-49] years). Of these, 603 patients underwent MABS (endoscopic, endoscopy-assisted, or robot-assisted procedures in 289, 302, and 12 patients, respectively) and 1809 patients underwent CBS. The median follow-up time was 84 months (93 in the MABS group and 80 months in the CBS group). Intergroup differences were not significant for the following parameters: 10-year local recurrence-free survival (93.3% vs 96.3%; hazard ratio [HR], 1.39; 95% CI, 0.86-2.27; *P* = .18), regional recurrence-free survival (95.5% vs 96.7%; HR, 1.38; 95% CI, 0.81-2.36; *P* = .23), and distant metastasis-free survival (81.0% vs 82.0%; HR, 0.95; 95% CI, 0.74-1.23; *P* = .72). The 5-, 10-, and 15-year disease-free survival rates in the MABS group were 85.9%, 72.6%, and 69.1%, respectively. The corresponding rates in the CBS group were 85.0%, 76.6%, and 70.7%. The intergroup differences were not significant (HR, 1.07; 95% CI, 0.86-1.31; *P* *=* .55). The 5-, 10-, and 15-year overall survival rates in the MABS group were 92.0%, 83.7%, and 83.0%, respectively. The corresponding rates in the CBS group were 93.6%, 88.7%, and 81.0%. The intergroup differences were not significant (HR, 1.29; 95% CI, 0.97-1.72; *P* *=* .09). Post hoc subgroup analysis showed no significant intergroup differences in disease-free survival.

**Conclusions and Relevance:**

In this cohort study, long-term outcomes following MABS were not significantly different from those following CBS in patients with early-stage breast cancer. MABS may be a safe and feasible alternative in this patient population.

## Key Points

**Question:**

What is the long-term oncologic prognosis associated with minimal access breast surgery (endoscopic or robotic) vs conventional breast surgery?

**Findings:**

This cohort study including 2412 patients with breast cancer found that the long-term oncologic prognosis following minimal access breast surgery was not significantly different than that following conventional breast surgery.

**Meaning:**

The findings indicate that minimal access breast surgery may be a safe and feasible alternative to conventional surgery.

## Teaser

This cohort study evaluates the association of oncologic prognosis with minimal access breast surgery vs conventional surgery.

## Introduction

The Global Cancer Statistics 2020 report^1^ showed an estimated 19.3 million new cancer cases worldwide, 9.23 million of which were in women, and 2.26 million of which were breast cancer in female individuals. Breast cancer has surpassed lung cancer as the foremost cause of cancer incidence and is the fifth leading cause of cancer-related death worldwide. A meta-analysis^2^ of global and regional breast cancer survival rates showed the global 1-, 3-, 5-, and 10-year pooled survival rates for women with breast cancer were 92%, 75%, 73%, and 61%, respectively. Breast cancer requires comprehensive treatment with surgery as the main treatment, combined with radiotherapy, chemotherapy, and endocrine and targeted therapies. Over the past few decades, due to the development of endoscopic, robotic, and reconstructive surgical procedures,^3,4,5,6^ the focus of advances in surgical technology has shifted to the maintenance of form, function, and quality of life.^7^

Endoscopic and robotic surgery have the advantage of low intraoperative blood loss, short hospital stay, and fast functional recovery. These methods have been widely used in gynecology, gastrointestinal surgery, urology, cardiothoracic surgery, and other disciplines.^8,9,10,11^ In breast surgery, endoscopic and robotic surgery developed relatively late compared with other disciplines. Studies^12,13,14,15,16,17,18,19,20,21^ have reported on the feasibility, cosmetic effects, and safety of endoscopic surgery and robotic surgical techniques in breast cancer. These techniques afford the advantages of concealed incisions, preserved function, reduced pain, and short postsurgery recovery time, all of which are associated with improved quality of life. In addition, no differences in overall survival (OS) and disease-free survival (DFS) have been reported between minimally invasive and conventional breast surgical (CBS) methods. Endoscopic and robotic breast surgery have been applied in studies on minimal access breast surgery (MABS),^14^ robotic nipple-sparing mastectomy,^15^ mastoscopic axillary lymph node dissection,^17^ endoscopic-assisted nipple-sparing mastectomy,^22^ endoscopy-assisted breast conservation surgery,^23^ and laparoscopy-assisted breast reconstruction.^24^

However, the efficacy of these techniques is controversial. Ramirez et al^25^ conducted studies on early cervical cancer stages IA1, IA2, and IB1 and reported that the 3- and 4.5-year DFS rates and 3-year OS rates in the minimally invasive surgery group were significantly lower than those in the open surgery group. In 1 epidemiological study,^26^ the mortality rate associated with minimally invasive surgery was significantly higher than that associated with open surgery in patients with cervical cancer stage IA2 or IB1 (hazard ratio [HR], 1.65; 95% CI, 1.22-2.22; *P* = .002). A meta-analysis^27^ showed that patients who underwent minimally invasive radical hysterectomy had a 71% higher pooled hazard risk of recurrence or death and a 56% higher risk of death than those who underwent open surgery. Multiple studies have reported that minimally invasive radical hysterectomy was associated with a lower survival rate and higher risks of recurrence and death than open abdominal radical hysterectomy in patients with early cervical cancer. These findings have brought into question the safety of minimally invasive surgery.^28,29^ In addition, most previous studies on breast surgery were about a single surgical procedure with short follow-up time. To our knowledge, there has not been a comprehensive long-term oncology safety and efficacy study comparing multiple surgical modalities of MABS and CBS.

On the basis of previous research at our center,^30,31,32,33^ this propensity score–matched retrospective cohort study aimed at comparing oncological outcomes following endoscopic breast surgery and robotic breast surgery with those following CBS and to explore long-term oncologic safety of these approaches.

## Methods

### Ethical Review

This study was approved by the Clinical Research Ethics Committee of Southwest Hospital, the First Affiliated Hospital of the Army Medical University, Chongqing, China (approval number: KY2021070). An exemption of informed consent was obtained owing to the retrospective nature of the data.

### Study Design and Patients

In this single-center retrospective cohort study, 9184 patients with breast cancer from Southwest Hospital, the First Affiliated Hospital of the Army Medical University, Chongqing, China, were evaluated for inclusion from January 1, 2004, to December 31, 2017. Data on patients’ demographic characteristics, preoperative imaging examinations, surgery, and preoperative and postoperative treatments as well as other related information were collected.

The inclusion criteria were female sex, age 18 to 75 years, disease stages 0 to III, unilateral surgery, no distant metastasis, and no history of breast cancer or severe underlying disease before surgery. Surgical procedures that patients had undergone included modified radical mastectomy, subcutaneous gland excision, simple mastectomy, breast-conserving surgery, breast reconstruction with CBS, endoscopic breast surgery, and robotic breast surgery.^18,21,30,31,34,35,36,37,38^ The exclusion criteria were incomplete basic information, receipt of surgery or chemotherapy in other hospitals, bilateral surgery, no surgery, receipt of other surgical methods, preoperative distant metastasis, younger than 18 years or older than 75 years, other concomitant surgical procedures, male sex, and receipt of intraoperative radiotherapy.

Total mastectomy was defined as modified radical mastectomy, subcutaneous gland excision, and simple mastectomy, while breast-conserving surgery was defined as partial mastectomy. Patients who underwent endoscopic, endoscopy-assisted, and robotic-assisted procedures were classified as the MABS group and those who underwent CBS as the CBS group. Pathological staging was per the eighth edition of the American Joint Committee on Cancer (AJCC) TNM staging system for breast cancer. Preoperative and postoperative treatments were conducted per breast cancer treatment guidelines, which recommended standard regimens and treatment courses.

### Propensity Score Matching

The propensity score,^39,40^ which is the conditional probability of being treated under the covariate condition, can reduce bias and equalize confounding factors between groups. The propensity score covariates in this study included age at diagnosis, lymph node status, pathological stage and type, hormone receptor status, ERBB2 (formerly HER2 or HER2/neu) status, and preoperative and postoperative chemotherapy. The propensity score was calculated by logistic regression analysis using the R software MatchIt package (R Foundation) and 1:3 nearest-neighbor matching without a caliper value. After matching, *P* values for the group samples were all greater than .05, indicating a good balance. A total of 603 and 1809 patients were included in the MABS and CBS groups, respectively.

### Follow-up

Follow-up started on the date of surgery and ended on December 31, 2019. Follow-up was conducted every 3 months for the first year, biannually from 1 to 5 years, and annually after 5 years. The follow-up methods included reexamination of outpatients and inpatients with medical records and telephonic follow-up. Recurrence and metastasis were defined according to whether either was determined based on computed tomography, magnetic resonance imaging, positron emission computed tomography, breast ultrasonography, or pathological biopsy findings and other data collected at the time of the patient’s visit. Death was defined as receipt of a death certificate from the hospital, and the date of death was recorded based on the information provided by the patient’s family during the telephonic follow-up.

### Study Outcomes

OS was defined as the time from the surgery date to death from any cause or the last follow-up. DFS was defined as the time from the surgery date to recurrence, metastasis, and death from any cause. Local recurrence was defined as recurrence in the ipsilateral chest wall and at the breast surgical site. Regional recurrence was defined as recurrence in the ipsilateral axillary, internal mammary, or supraclavicular lymph node drainage area. Recurrence at any other side was considered to be distant metastasis. If 2 or more events occurred simultaneously, they were counted individually. Local recurrence-free survival was defined as the time from the surgery date to local recurrence. Regional recurrence-free survival was the time from the surgery date to regional recurrence. Distant metastasis-free survival was defined as the time from the surgery date to metastasis to distant sites. The primary study end points were local recurrence-free survival, regional recurrence-free survival, distant metastasis-free survival, DFS, and OS.

### Statistical Analysis

Survival curves were estimated by the Kaplan-Meier method for local recurrence-free survival, regional recurrence-free survival, distant metastasis-free survival, DFS, and OS, the log-rank test was used for between-group comparisons. The median follow-up time was estimated by the reverse Kaplan-Meier method. Cox proportional hazards models were used to estimate HRs and 95% CIs, and univariate and multivariate analyses were performed. Categorical variables were compared using the χ^2^ or Fisher exact test. Continuous variables were compared using a *t* test. A 2-tailed *P* value <.05 was considered significant. R version 4.1.2 (R Foundation) and SPSS version 26 (IBM) were used for analyses.

## Results

**Figure 1.**
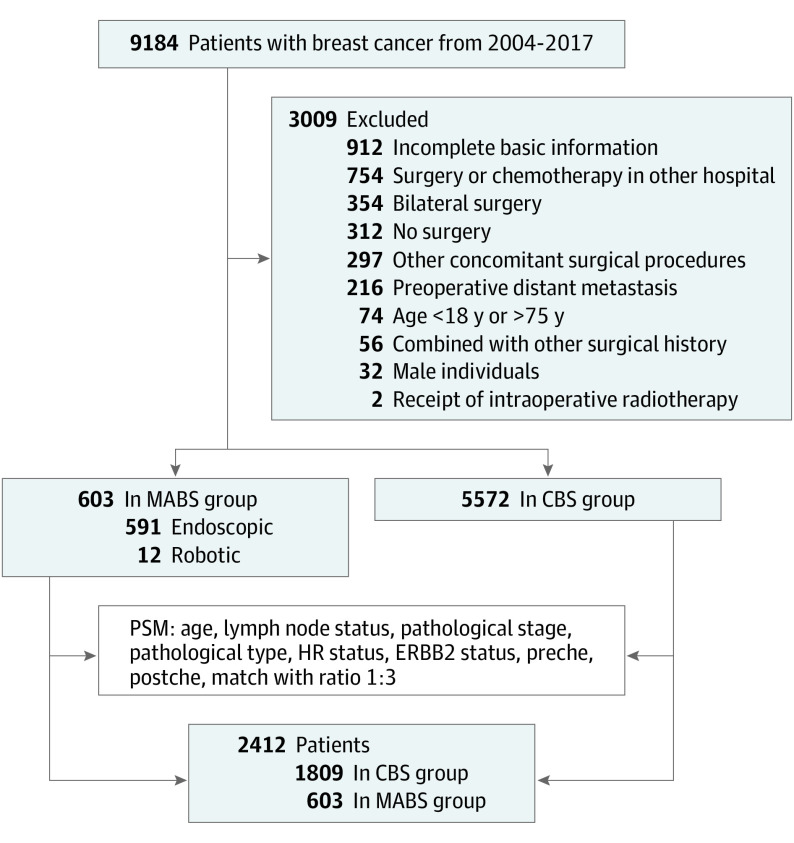
Flow Diagram of Participant Selection CBS indicates conventional breast surgery; MABS, minimal access breast surgery; PSM, propensity score matching.

A total of 6175 patients with breast cancer were considered eligible for this study: 603 (9.77%) in the MABS group (including 289 [47.93%], 302 [50.08%], and 12 [1.99%] received endoscopic breast surgery, endoscopic-assisted breast surgery, and robotic-assisted breast surgery, respectively) and 5572 (90.23%) in the CBS group. After propensity score matching, 2412 patients were included in the study (100% female; median [IQR] age, 44 [40-49] years), including 603 in the MABS group and 1809 in the CBS group (Figure 1). Table 1 summarizes the selected demographic and clinical characteristics of the study population before and after propensity score matching. Before propensity score matching, the groups showed no significant differences in pathological type, hormone receptor status, or ERBB2 status. However, they showed statistically significant differences in age at diagnosis, lymph node status, pathological stage, preoperative chemotherapy, and postoperative chemotherapy. After matching, the groups showed no significant differences in age at diagnosis, lymph node status, pathological stage, pathological type, hormone receptor status, ERBB2 status, preoperative chemotherapy and postoperative chemotherapy, and the baseline values of the 2 groups were the same.

**Table 1.** Baseline Characteristics of Patients Before and After Propensity Score Matching (PSM)

Characteristic	Before PSM, No. (%)			After PSM, No. (%)		
	MABS (n = 603)	CBS (n = 5572)	*P* value	MABS (n = 603)	CBS (n = 1809)	*P* value
No.	603	5572	NA	603	1809	NA
Age, median (IQR), y	43.0 (38.5-49.0)	47.0 (42.0-54.0)	NA	43.0 (38.5-49.0)	45.0 (40.0-49.0)	NA
Age, y						
<50	454 (75.29)	3316 (59.51)	<.001	454 (75.29)	1360 (75.18)	>.99
≥50	149 (24.71)	2256 (40.49)		149 (24.71)	449 (24.82)	
Lymph node status						
Negative	386 (64.01)	3216 (57.72)	.003	386 (64.01)	1150 (63.57)	.88
Positive	217 (35.99)	2356 (42.28)		217 (35.99)	659 (36.43)	
Cancer stage						
0-I	242 (40.13)	1971 (35.37)	.03	242 (40.13)	714 (39.47)	.93
II	273 (45.27)	2611 (46.86)		273 (45.27)	821 (45.38)	
III	88 (14.59)	990 (17.77)		88 (14.59)	274 (15.15)	
Pathology						
Noninvasive	24 (3.98)	237 (4.25)	.83	24 (3.98)	57 (3.15)	.40
Invasive	579 (96.02)	5335 (95.75)		579 (96.02)	1752 (96.85)	
Hormone receptor status						
Negative	161 (26.70)	1695 (30.42)	.07	161 (26.70)	521 (28.80)	.35
Positive	442 (73.30)	3877 (69.58)		442 (73.30)	1288 (71.20)	
ERBB2 status						
Negative	400 (66.33)	3656 (65.61)	.08	400 (66.33)	1176 (65.01)	.64
Positive	161 (26.70)	1377 (24.71)		161 (26.70)	486 (26.87)	
Unsure	42 (6.97)	539 (9.67)		42 (6.97)	147 (8.13)	
Preoperative chemotherapy						
No	165 (27.36)	3208 (57.57)	<.001	165 (27.36)	486 (26.87)	.85
Yes	438 (72.64)	2364 (42.43)		438 (72.64)	1323 (73.13)	
Postoperative chemotherapy						
No	50 (8.29)	935 (16.78)	<.001	50 (8.29)	147 (8.13)	.97
Yes	553 (91.71)	4637 (83.22)		553 (91.71)	1662 (91.87)	
Lymph node surgery						
SLNB	69 (11.44)	788 (14.14)	.08	69 (11.44)	191 (10.56)	.60
ALND	534 (88.56)	4784 (85.86)		534 (88.56)	1618 (89.44)	
Breast surgery						
Partial	80 (13.27)	859 (15.42)	.18	80 (13.27)	255 (14.10)	.66
Total	523 (86.73)	4713 (84.58)		523 (86.73)	1554 (85.90)	

Abbreviations: ALND, axillary lymph node dissection; CBS, conventional breast surgery; MABS, minimal access breast surgery; NA, not applicable; SLNB, sentinel lymph node biopsy.

At the study follow-up deadline, the median (IQR) follow-up time for all patients was 84 (45-112) months (range, 0-91 months; 95% CI, 81-87) months in the MABS group, and 80 (45-110) months in the CBS group. The lost to follow-up rate was 15.9% (96 of 603) in the MABS group and 13.0% (235 of 1809) in the CBS group. The mortality rate was 11.1% (67 of 603) in the MABS group and 8.3% (151 of 1809) in the CBS group.

**Figure 2.**
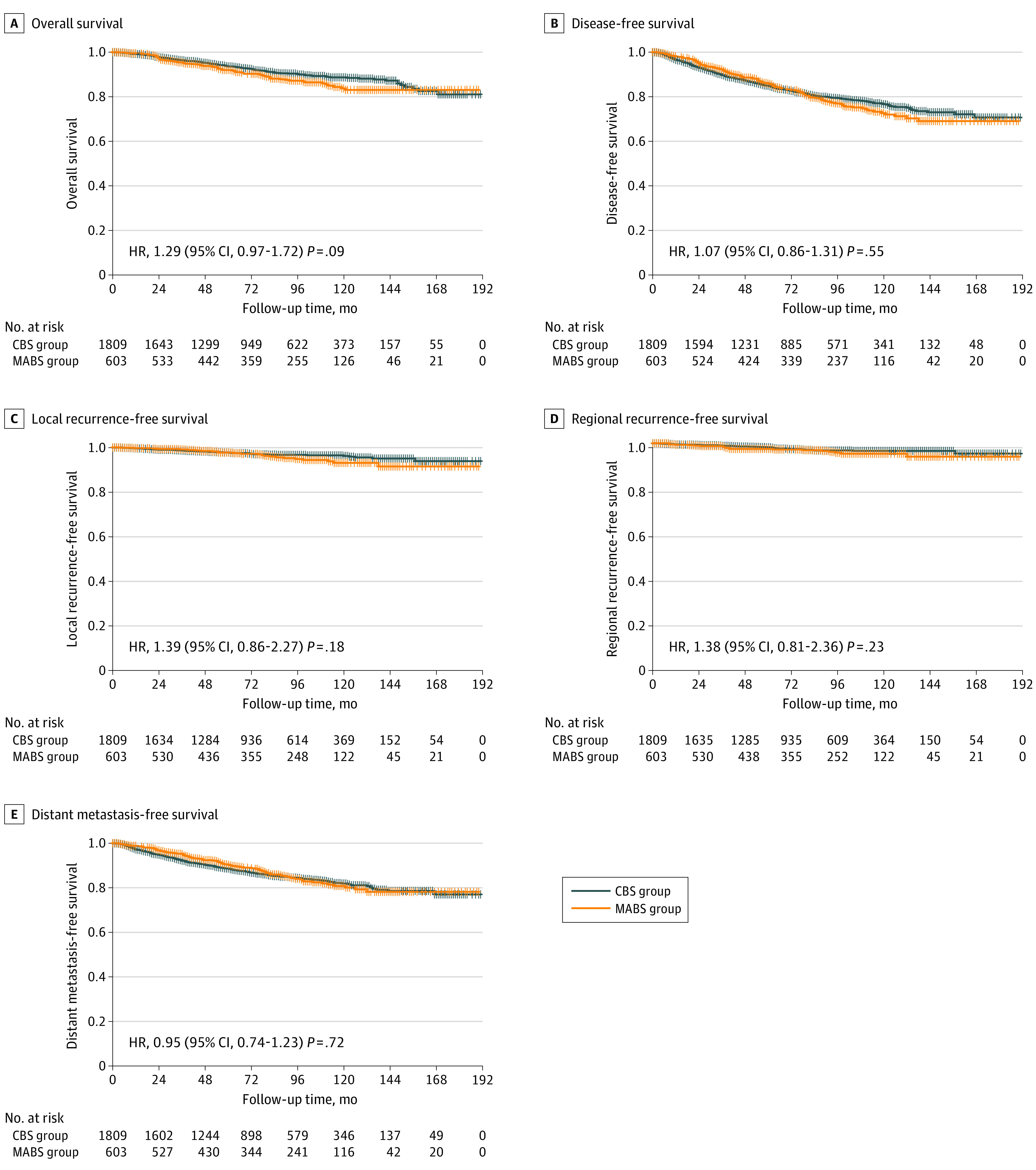
Kaplan-Meier Survival Curve After Propensity Score Matching Propensity score–matched Kaplan-Meier survival analysis showing no significant differences between the MABS and CBS groups in overall survival, disease-free survival, local recurrence-free survival, regional recurrence-free survival, or distant metastasis-free survival.

The MABS and CBS groups showed no significant differences in rates of local recurrence (4.0% vs 2.8%; *P* = .13), regional recurrence (3.3% vs 2.3%; *P* = .18), and distant metastasis (13.4% vs 13.5%; *P* = .95). Figure 2 shows the long-term survival results. The MABS and CBS groups showed no significant differences in the 10-year local recurrence-free survival (93.3% vs 96.3%; HR, 1.39; 95% CI, 0.86-2.27; *P* = .18) or regional recurrence-free survival (95.5% vs 96.7%; HR, 1.38; 95% CI, 0.81-2.36; *P* = .23). Distant metastasis-free survival (81.0% vs 82.0%; HR, 0.95; 95% CI, 0.74-1.23; *P* = .72). The 5-year DFS was 85.9% in the MABS group and 85.0% in the CBS group, 10-year DFS was 72.6% in the MABS group and 76.6% in the CBS group, and 15-year DFS was 69.1% in the MABS group and 70.7% in the CBS group, with no significant intergroup differences (HR, 1.07; 95% CI, 0.86-1.31; *P* = .55). The 5-year OS was 92.0% in the MABS group and 93.6% in the CBS group, 10-year OS was 83.7% in the MABS group and 88.7% in the CBS group, and 15-year OS was 83.0% in the MABS group and 81.0% in the CBS group, which were still not statistically significant (HR, 1.29; 95% CI, 0.97-1.72; *P* = .09).

In the Cox univariate and multivariate analysis of DFS and OS, the univariate analysis showed that lymph node status, pathological stage, pathological type, hormone receptor status, ERBB2 status, preoperative chemotherapy, postoperative chemotherapy, and lymph node surgery may be associated with the prognosis of DFS and OS. Table 2 shows the results for the multivariate analysis of DFS after the Cox proportional hazards model test. The differences were statistically significant for lymph-node positive status (HR, 1.85; 95% CI, 1.39-2.46; *P* < .001), pathological stage II (HR, 1.37; 95% CI, 1.01-1.85; *P* = .045), pathological stage III (HR, 2.95; 95% CI, 2.02-4.32; *P* < .001), ERBB2 positive status (HR, 1.37; 95% CI, 1.16-1.68; *P* = .003), and preoperative chemotherapy (HR, 1.32; 95% CI, 1.05-1.67; *P* = .02). However, no significant differences were observed in age at diagnosis, pathological type, hormone receptor status, postoperative chemotherapy, lymph node surgery, and breast surgery. In the multivariate analysis of OS (Table 2), lymph node-positive status (HR, 2.91; 95% CI, 1.86-4.55; *P* < .001), pathological stage III and II (HR, 2.21; 95% CI, 1.25-3.91; *P* = .006), hormone receptor status (HR, 0.56; 95% CI, 0.42-0.74; *P* < .001), preoperative chemotherapy (HR, 1.70; 95% CI, 1.18-2.45; *P* = .004), the difference was statistically significant. In contrast, there were no significant differences in age at diagnosis, pathological type, ERBB2 status, postoperative chemotherapy, lymph node surgery, and breast surgery. Most importantly, in the univariate analysis, no significant difference was observed between the MABS and CBS groups in terms of DFS (HR, 1.07; 95% CI, 0.86-1.31; *P* = .55) or OS (HR, 1.29; 95% CI, 0.97-1.72; *P* = .09).

**Table 2.** Univariate and Multivariate Analysis With Disease-Free Survival and Overall Survival

Variable	Disease-free survival				Overall survival			
	Univariate analysis, HR (95% CI)	*P* value	Multivariate analysis, HR (95% CI)	*P* value	Univariate analysis, HR (95% CI)	*P* value	Multivariate analysis, HR (95% CI)	*P* value
Age, y								
<50	1 [Reference]	NA	1 [Reference]	NA	1 [Reference]	NA	1 [Reference]	NA
≥50	0.91 (0.74-1.13)	.41	NA	NA	0.87 (0.64-1.18)	.37	NA	NA
Lymph node status								
Negative	1 [Reference]	NA	1 [Reference]	NA	1 [Reference]	NA	1 [Reference]	NA
Positive	3.33 (2.74-4.04)	<.001	1.85 (1.39-2.46)	<.001	4.06 (3.04-5.43)	<.001	2.91 (1.86-4.55)	<.001
Cancer stage								
0-I	1 [Reference]	NA	1 [Reference]	NA	1 [Reference]	NA	1 [Reference]	NA
II	1.95 (1.52-2.51)	<.001	1.37 (1.01-1.85)	.045	1.58 (1.19-2.10)	.002	1.02 (0.63-1.65)	.93
III	5.83 (4.50-7.54)	<.001	2.95 (2.02-4.32)	<.001	1.26 (0.76-2.09)	.37	2.21 (1.25-3.91)	.006
Pathology								
Noninvasive	1 [Reference]	NA	1 [Reference]	NA	1 [Reference]	NA	1 [Reference]	NA
Invasive	2.58 (1.15-5.77)	.02	NA	NA	3.74 (0.93-15.04)	.06	NA	NA
Hormone receptor status								
Negative	1 [Reference]	NA	1 [Reference]	NA	1 [Reference]	NA	1 [Reference]	NA
Positive	0.87 (0.71-1.07)	.19	NA	NA	0.72 (0.55-0.96)	.02	0.56 (0.42-0.74)	<.001
ERBB2 status								
Negative	1 [Reference]	NA	1 [Reference]	NA	1 [Reference]	NA	1 [Reference]	NA
Positive	1.55 (1.27-1.88)	<.001	1.37 (1.16-1.68)	.003	1.58 (1.19-2.10)	.002	NA	NA
Unsure	1.03 (0.71-1.50)	.87	1.12 (0.77-1.63)	.54	1.26 (0.76-2.09)	.37	NA	NA
Preoperative chemotherapy								
No	1 [Reference]	NA	1 [Reference]	NA	1 [Reference]	NA	1 [Reference]	NA
Yes	1.47 (1.17-1.84)	.001	1.32 (1.05-1.67)	.02	2.13 (1.48-3.06)	<.001	1.70 (1.18-2.45)	.004
Postoperative chemotherapy								
No	1 [Reference]	NA	1 [Reference]	NA	1 [Reference]	NA	1 [Reference]	NA
Yes	1.57 (1.01-2.43)	.04	NA	NA	1.97 (0.97-4.00)	.06	NA	NA
Lymph node surgery								
SLNB	1 [Reference]	NA	1 [Reference]	NA	1 [Reference]	NA	1 [Reference]	NA
ALND	2.28 (1.48-3.50)	<.001	NA	NA	2.96 (1.46-5.99)	.003	NA	NA
Breast surgery								
Partial	1 [Reference]	NA	1 [Reference]	NA	1 [Reference]	NA	1 [Reference]	NA
Total	1.30 (0.98-1.72)	.07	NA	NA	1.44 (0.95-2.19)	.09	NA	NA
Group								
CBS	1 [Reference]	NA	1 [Reference]	NA	1 [Reference]	NA	1 [Reference]	NA
MABS	1.07 (0.86-1.31)	.55	NA	NA	1.29 (0.97-1.72)	.09	NA	NA

Abbreviations: ALND, axillary lymph node dissection; CBS, conventional breast surgery; HR, hazard ratio; MABS, minimal access breast surgery; NA, not applicable; SLNB, sentinel lymph node biopsy.

**Figure 3.**
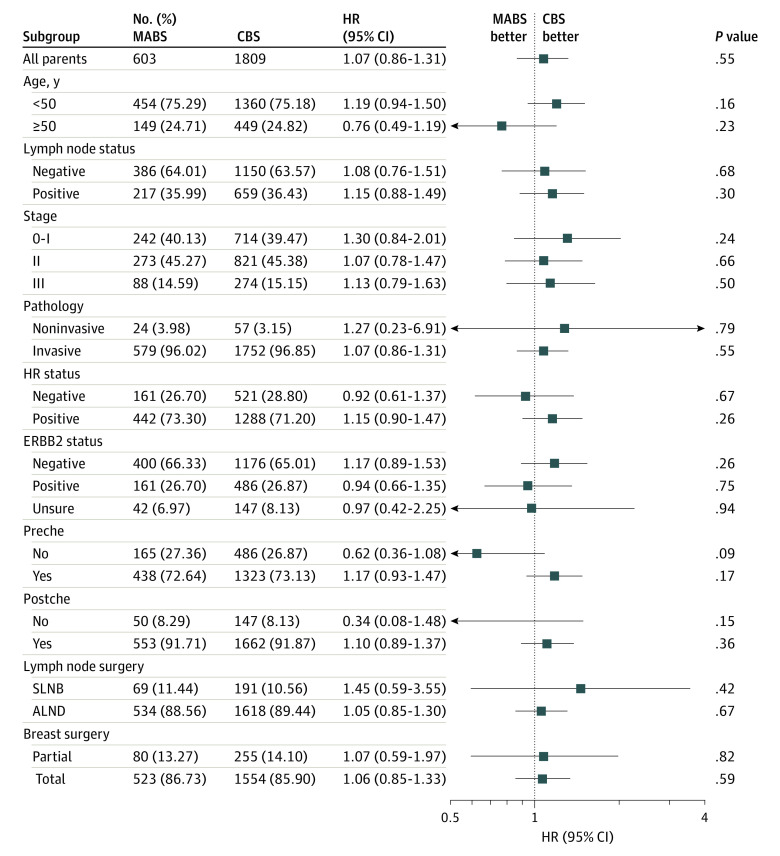
Forest Plot for Subgroup Analysis of Disease-Free Survival Forest plot of subgroup analysis of distant metastasis-free survival showing no significant differences by subgroup.

Exploratory post hoc subgroup analysis was performed on the matched data, with subgroup analysis of DFS (Figure 3), age at diagnosis (younger than 50 years and 50 years and older), lymph node status (negative and positive), pathological stage (0-I, II, and III), pathological type (noninvasive and invasive), hormone receptor status (negative and positive), ERBB2 status (negative, positive, and unsure), preoperative chemotherapy (no and yes), and postoperative chemotherapy (no and yes), lymph node surgery (sentinel lymph node biopsy and axillary lymph node dissection), and breast surgery (partial and total), and the results showed no significant differences in all subgroup analyses between the 2 groups.

## Discussion

To our knowledge, this is the first propensity-matched retrospective cohort study to compare long-term outcomes following MABS vs CBS. The study had a longer follow-up time and included multiple minimal access surgical methods and surgical procedures. During follow-up, the MABS and CBS groups showed no significant difference in local recurrence-free survival, regional recurrence-free survival, distant metastasis-free survival, DFS, or OS in patients with stage 0 to III breast cancer. The univariate analysis also indicated no significant association between surgical procedures and DFS or OS. This finding differs from the results of previous studies in which individuals with early cervical cancer who underwent minimally invasive surgery showed a higher risk of recurrence and mortality than those who underwent open surgery.

Before propensity score matching, the CBS group was older, had more positive lymph nodes, and was at a later cancer stage. Preoperative and postoperative chemotherapy was administered to a greater percentage of patients in the MABS group. After matching, the 2 groups showed no significant difference in terms of age at diagnosis, lymph node status, pathological type, hormone receptor status, ERBB2 status, pathological stage, preoperative chemotherapy, or postoperative chemotherapy, and their baseline values were nearly identical.

The 2 groups showed no statistically significant difference in DFS or OS in this study. Luo et al^16^ found no significant difference in DFS (64.5% vs 60.8%; *P* = .88) and OS (81.7% vs 78.6%; *P* = .95) between groups undergoing mastoscopic axillary lymph node dissection vs completion axillary lymph node dissection. Lai et al^41^ reported a difference in OS between endoscope-assisted breast surgery and conventional surgery groups before propensity score matching but this difference was no longer significant after propensity score matching. In a comparison^30^ of endoscopic nipple-sparing mastectomy and immediate implant reconstruction with breast-conserving surgery, DFS and OS in the breast-conserving surgery group were lower than those in the endoscopic-assisted nipple-sparing mastectomy group, but the difference did not show statistical significance. In a prospective study^15^ on robotic nipple-sparing mastectomy, 94 patients underwent robotic nipple-sparing mastectomy with a median (range) follow-up period of 19 (3.1-44.8) months. There were no recurrences in the study; the OS at 12, 24, and 60 months was 98% (95% CI, 86-100); and the differences in OS and DFS were not statistically significant. The results of these studies are consistent with the results of our study.

In the univariate analysis in this study, surgical modality was not an influencing factor for OS and DFS (Table 2). The report also indicated that minimally invasive breast surgery was not associated with the risk of disease recurrence in a multivariate analysis of disease recurrence.^14^ Common factors associated with risk for OS and DFS prognosis in multivariate analysis were lymph node metastases, pathological stage, and preoperative chemotherapy. Patients with positive hormone receptor status showed a lower chance of OS than those with negative hormone receptor status, while those with positive ERBB2 status showed a higher risk of DFS than those with negative ERBB2 status. A series of reports^42,43,44,45^ indicated that factors associated with prognosis included tumor size, number of lymph node metastases, tumor type, tumor stage, histological grade, estrogen receptor and progesterone receptor status, chemotherapy, and antiestrogens. These were similar to the factors associated with prognosis in the multivariate analysis in our study.

When an exploratory post hoc subgroup analysis was performed on the matched data, the DFS analysis in each subgroup showed no statistically significant difference between the 2 groups (Figure 3). Similarly, the difference in DFS among all patients was not statistically significant.

### Limitations

This study has limitations. First, we did not account for hidden confounding factors. The CBS and MABS groups also showed discrepancies in terms of median follow-up time and mortality rate. Since the CBS group had more data than the MABS group, patients in the CBS group may have had their data preferentially matched from recent years, but this did not alter the interpretation of the results. Additionally, this was a retrospective study, and although the propensity score matching method was used to reduce bias between the 2 groups, the possibility of selection bias could not be eliminated entirely. In the future, multicenter prospective large-sample studies with longer follow-up periods will be required to confirm the long-term oncological safety of MABS.

## Conclusions

The findings of this study revealed no statistically significant differences in DFS, OS, local recurrence-free survival, regional recurrence-free survival, and distant metastasis-free survival among the long-term oncological outcomes between the MABS and CBS groups, and also showed no differences between the 2 surgical procedures. MABS provides the advantages of less trauma, good cosmetic results, functional preservation, and high patient satisfaction. Furthermore, the surgical approach was not associated with prognosis in our investigation, and long-term oncological outcomes were not significantly different. For patients with early-stage breast cancer, MABS may offer a valid alternative surgical option.
